# Editorial for the Special Issue on Multimodality and Sequential Therapy in Locally Advanced Head and Neck Cancer

**DOI:** 10.3390/cancers14174247

**Published:** 2022-08-31

**Authors:** Giuseppe Riva, Giancarlo Pecorari

**Affiliations:** Division of Otorhinolaryngology, Department of Surgical Sciences, University of Turin, 10126 Turin, Italy

Multimodal and sequential treatment for locally advanced head and neck cancer (HNC) included induction chemotherapy, chemoradiation organ preservation protocols, immunotherapy, and targeted therapy. Definitive management of locally advanced HNC is evolving into a tailored therapy according to the patient’s risk. Besides survival, organ function preservation became an important goal of HNC treatment. The combination of chemotherapy, radiotherapy, targeted therapy, and less demolitive surgical procedures allowed for the performance of organ and function preservation [1]. This Special Issue of Cancers, entitled “Multimodality and Sequential Therapy in Locally Advanced Head and Neck Cancer”, contains five papers (three reviews and two research articles) that highlight recent advances in multimodal and sequential treatment for locally advanced HNC. The papers can be categorized as follows:

1. Preclinical study: HPV-positive HNC has a better prognosis after chemoradiotherapy. Subtil et al. analyzed the effect of the dual PI3K/mTOR inhibitor NVP-BEZ235 on a combined treatment with cisplatin and radiation in six HPV-negative and six HPV-positive HNC cell lines. Solely for HPV-positive cells, pretreatment with BEZ235 resulted in enhanced cisplatin sensitivity. Moreover, when pretreated with BEZ235, the combination of cisplatin and radiation changed into a synergistic interaction, with a slightly stronger enhancement for HPV-positive cells [2].
2. Prognostic role of imaging findings: Martens et al. showed that intratreatment functional imaging parameters, obtained by MRI and 18F-FDG-PET/CT, captured early tumoral changes that provided prognostic information regarding locoregional recurrence-free survival [3]. On the other hand, the systematic review by Russo et al. on oropharyngeal squamous cell carcinoma demonstrated that primary and nodal tumor volume seemed not to behave as reliable prognostic factors [4].
3. Surgery: Accorona et al. performed a systematic review on free periosteal flaps with scaffold for maxillary and mandibular reconstruction. The literature reported an overall rate of complications of 43.7% and a success rate, intended as scaffold integration, of 74.5%. The authors concluded that such a technique may represent an alternative for patient unfit for complex bone free flaps reconstruction [5].
4. Chemoradiation therapy: treatment-related toxicity negatively affects patients’ quality of life. A literature review on sinonasal side effects of chemotherapy and/or radiation therapy for HNC by Riva et al. showed that the incidence and severity of olfactory dysfunction and chronic rhinosinusitis were highest at the end of radiotherapy and at three months after treatment and decreased gradually over time. These side effects seemed to be related to radiation dose on the olfactory area and nasal cavities, but different degrees of recovery were observed. Therefore, it became important to establish the severity of chronic rhinosinusitis and olfactory dysfunction in order to find strategies to support patients and improve their quality of life [6].

This Special Issue highlighted how important are both preclinical and clinical studies to improve the oncologic and functional outcomes of HNC treatments. Further studies would analyze the combination of different therapies for locally advanced HNC.

We would like to thank all the authors for their submissions to this Special Issue. We also thank all the reviewers for dedicating their time and helping ensure the quality of the submitted papers, and, last but not least, the staff at the editorial office of Cancers for their efficient assistance.
